# Norcantharidin Nanostructured Lipid Carrier (NCTD-NLC) Suppresses the Viability of Human Hepatocellular Carcinoma HepG2 Cells and Accelerates the Apoptosis

**DOI:** 10.1155/2022/3851604

**Published:** 2022-04-20

**Authors:** Zijun Yan, Kun Yang, Xiang Tang, Yunfeng Bi, Yuzhen Ding, Mengyue Deng, Die Xia, Yunqi Zhao, Tong Chen

**Affiliations:** ^1^School of Pharmaceutical Sciences and Yunnan Key Laboratory of Pharmacology for Natural Products, Kunming Medical University, Yunnan, Kunming 650500, China; ^2^Department of Pharmacy, Panzhihua Central Hospital, Sichuan, Panzhihua 617067, China; ^3^School of Pharmacy, Dali University, Yunnan, Dali 671000, China; ^4^College of Science and Technology, Wenzhou-Kean University, Zhejiang, Wenzhou 325060, China

## Abstract

Malignant tumors have become the main cause of harm to human life and health. Development for new antitumor drugs and the exploration to drug carriers are becoming the concerned focus. In this study, we exploited our experiments to explore the effect of NCTD-NLC on liver cancer cells: the HepG2 cells cultured in vitro were given with NCTD-NLC administration; then, the estimation on cellular proliferation and apoptosis was accomplished through MTT and flow cytometry. Six hours after the administration, we performed the High Performance Liquid Chromatography (HPLC) detection to estimate the NCTD content in the heart, liver, spleen, lung, kidney and plasma of rats. Then, our outcomes showed that NCTD-NLC had a notable inhibitory effect on HepG2 cells, leading to a gradually decreased cellular viability. Cell viability was negatively correlated with NCTD-NLC concentration. Along with the concentration increasing, significantly increasing cellular apoptosis and gradually decreasing cellular viability were observed. The apoptosis rate was positively correlated with the concentration of NCTD-NLC. On the basis of the data we obtained, we found that the group with NCTD-NLC tail vein injection had an obvious advantage in drug delivery when compared with other groups. Through the tumorigenesis test to nude mice, we found that the tumor inhibition rate of the NCTD-NLC tail vein injection group had a 27.48% elevation in contrast to the NCTD gavage group, and it was also the group with the best tumor inhibition efficiency. In conclusion, the NCTD-NLC prepared in this study had a mighty inhibitory effect towards HepG2 cellular viability and an accelerating work on apoptosis. Tail vein injection of NCTD-NLC has the best drug delivery effect.

## 1. Introduction

The high morbidity of malignancies is severely jeopardizing public health in recent year. Cancer is one of the primarily death causes around the world. Nowadays, liver cancer has ranked the sixth cancer death worldwide [1, 2]. Moreover, the survival rate of liver cancer in China is the worst. Conventional preparations are short in their half-life and bioavailability, so only a small amount of drug reaches the tumor site, increasing the dose of the drug but also increasing the toxic and side effects [3–5]. With the intention of improving the selectivity of the drugs and minimizing their toxicity, further research and development with biocompatibility and biodegradability were performed to attain the purpose of slow drug release, which would not only fortify the effect of drug targeting but also enable the drug treatment to achieve synergy and detoxification. The exploration to drug carriers of high molecular material is becoming a hot research topic at present.

Norcantharidin (NCTD) is a derivative of cantharidin. It is the first new drug developed in my country to treat liver cancer and hepatitis. NCTD could increase white blood cells, protecting liver cells and regulating immunity. It is mainly used for primary liver cancer treatment. Due to the lack of 2 methyl groups at positions 1 and 2 of cantharidin, NCTD reduces the strong irritation to the urinary system and digestive system, and the whitening effect is more obvious [6, 7]. However, it has been found in clinical applications that although NCTD reduces the toxicity of cantharidin to a certain extent, it is still highly toxic to the urinary system and digestive system. The main reason is that NCTD is a supramolecular antitumor drug. After gavage of normathenin in mice for 15 minutes to 2 hours, NCTD was found in the liver, kidney, tumor, stomach, intestine, heart, and lung; after intravenous administration, it also has a high tissue distribution in the liver, gallbladder, kidney, heart, and lung. Therefore, its tumor targeting is poor, and it still has certain side effects on normal tissues. In addition, the clearance rate of NCTD in various organs of mice is relatively fast, and the amount of the drug remaining in the body after 6 hours of administration is very low, which affects the therapeutic effect of cancer. In conclusion, the nonspecific distribution in vivo and insufficient local residence time in the tumor are the main factors that limit the optimal efficacy of NCTD, and the targeted drug delivery system of NCTD has become a research hotspot [8, 9].

Solid lipid nanoparticle (SLN) is composed of sterically immiscible liquid lipid and solid lipid matrix, forming crystals with many defective structures for drug loading [10, 11]. However, the future applicability of this formulation is due to the low drug loading. Incorporating liquid lipids into the solid lipids of nanoparticles elevates the number of defects in the core solid lipids, thereby promoting the increased amount of drugs while maintaining the physical stability of the nanocarriers [12, 13]. As a modified lipid nanoparticle, NLC is produced from a mixture of solid lipids (long chains) and liquid lipids (short chains) versus SLN, and NLC has a lower melting point due to its oil content, while maintaining its granular character and being solid at body temperature. NLC systems also have the advantages of SLNs, such as controlled drug release, biocompatibility, and the possibility of large-scale production.

NCTD is a characteristic insect drug with antitumor effect first discovered in my country, but its serious toxicity and side effects, low drug loading, and poor targeting of existing delivery systems limit its clinical application [14, 15]. Based on the current research data, we put forward the following hypothesis: the nanostructured lipid carrier can improve the drug loading of NCTD and achieve controlled drug release and antitumor targeting. To determine the difference between the nanostructured lipid carrier-mediated NCTD supramolecular and the free drug NCTD and also to determine whether the nanostructured lipid carrier has tumor targeting effect, the above evaluation was carried out by establishing an animal model and simulating human pharmacodynamics. This study is aimed at clarifying the specific mechanism of its targeting after the nanostructured lipid carrier. Cancer drugs and new delivery systems will further transform traditional characteristic medicinal animal resources into fruit and find new breakthroughs.

## 2. Materials and Methods

### 2.1. NCTD-NLC Preparation

10.5 mg NCTD (Macklin, N837936, China), 1.6511 g ethyl oleate (Macklin, E808949, China), 0.6018 g cremophor (Macklin, c804845, China), 0.4521 g PEG-400 (Macklin, P815608, China), and 0.3 mL water were weighed and placed in a water bath at 40°C. The mixture was magnetically stirred for 3 hours, then placed in a water bath of 20°C for stirring. Slowly adding water to the preparation while stirring and then stirring in the dark 10 min, the NCTD-NE (W/O) preparation was obtained.

1.0 mL NCTD-NE (W/O), 127.0 mg glyceryl monostearate (Macklin, G831016, China), 102.1 mg lecithin (Macklin, l812366, China), 51.0 mg glyceryl tripalmitate (Macklin, G875503, China), 6.1 mg stearamide (Macklin, N829520, China), 131.0 mg Tween-80 (Macklin, T818928, China), 20.0 mL dichloromethane (Macklin, d807825, China), 5 mL water, and 20 mL dichloromethane were put it in a water bath at 40°C. The mixture was spun dry in the dark to form a light yellow film on the inner wall of the round-bottomed flask. Tween-80 was added and placed in an ice bath and sonicated for 8 min to obtain the NCTD-NLC milky suspension.

### 2.2. HepG2 Cell Culture and Drug Treatment

HepG2 cells were seeded in 96-well plates according to the conventional adherent cell culture method. Culture conditions were as follows: DMEM+10% fetal bovine serum (Gibco, 10099-141, USA), cultured in a constant temperature incubator with 5% CO_2_ and 37°C. During the experiment, tumor cells in the logarithmic growth phase were digested with trypsin, adjusted to 1 × 10^4^/well, inoculated in a 96-well plate, and then added to a new culture containing drugs after adhering to the wall. The final concentrations of NCTD-NLC were 25 μmol/L, 50 μmol/L, 100 μmol/L, and 200 μmol/L, respectively. At the same time, the free drug NCTD control group was set at 100 μmol/L and the NS control group, with 3 replicate wells in each group.

### 2.3. MTT to Detect the Proliferation of NCTD-NLC-Treated Liver Cancer HepG2 Cells

Experimental grouping and treatment are as follows: NS control group, NCTD control group 100 μmol/L, HepG2 cells+25 μmol/L NCTD-NLC, HepG2 cells+50 μmol/L NCTD-NLC, HepG2 cells+100 μmol/L NCTD-NLC, and HepG2 cells+200 μmol/L NCTD-NLC. MTT detection was performed after incubation for 24 h and 48 h.

Logarithmic growth phase of cells was digested and resuspended at a concentration of 1 × 10^5^ cells/mL, inoculated in 96-well plates, and 3 replicate wells for each group. The cells were grouped after adherence and placed in a carbon dioxide incubator. After 24 h and 48 h, 20 *μ*L MTT solution (Beyotime, ST316, China) was added to each well. Four hours incubation afterward, the medium was aspirated. 150 μl of dimethyl sulfoxide was added to each well. The absorbance of each well was measured at OD 490 nm [16].

### 2.4. Flow Cytometry Detection of HepG2 Cell Apoptosis after Drug Addiction

The cells were cultured in a 6-well plate, and the cells were processed according to the grouping requirements, and the culture was continued for 48 h. Cell culture medium was aspirated into a centrifuge tube, with the cells once washed with PBS; then, 1 mL of trypsin was added to digest the cells. Cells were incubated at room temperature until gently pipetting down to remove the trypsinized cells. About 1 × 10^5^ resuspended cells were taken and centrifuged at 2000 rpm for 3 min with the supernatant discarded; then, 195 *μ*L Annexin V-FITC binding solution was added into gently resuspend the cells. 5 *μ*L Annexin V-FITC was added and mixed gently. Cells were incubated at 25°C for 10 min in the dark. 10 *μ*L of PI staining solution was added, mixed gently, and placed in an ice bath away from light [17].

### 2.5. Animal Model Administration

Forty 8-week-old male SPF SD rats were used in this experiment. The animals were provided by the Experimental Animal Center of Kunming Medical University (license number: SCXK (Dian) 2015-0002).

The animals were kept free to drink water and eat at a temperature of 23-25°C. Animals were adaptively fed for one week and marked with numbers. The experiment was divided into four groups, with 10 rats in each group: group 1, the rats were given NCTD-NLC according to the clinical conversion dose; group 2, the rats were given NCTD-NLC by gavage; group 3, rats were given NCTD-NLC according to the clinical conversion dose and administered by tail vein injection; and group 4, rats were given NCTD according to the clinical conversion dose and administered by tail vein injection.

The clinical dosage of NCTD is 15-45 mg/person/day, and the median value is 30 mg/person/day; according to Xu Wensheng's formula, the dose administered to rats is 163 mg/kg. The NCTD solution was administered at 32.6 mg/mouse per mouse. Each animal was given 200 *μ*L tail vein injection (drug concentration 163 mg/mL) and 2 mL intragastric administration (drug concentration 16.3 mg/mL); NCTD-NLC was given the same dose. After the experiment, the rats were anesthetized by intraperitoneal injection of 7% chloral hydrate (5 mL/kg) to collect the samples.

### 2.6. HPLC Detection of NCTD Content in Rat Heart, Liver, Spleen, Lung, Kidney, and Plasma

Samples were homogenized with a rapid homogenizer and were centrifuged at 10,000 r/min for 5 min. 200 *μ*L of supernatant tissue homogenate was collected. Add 3 mL of methanol solution then centrifuged at 10000 r/min for 5 min. The protein-depleted supernatant was collected and filtered at 0.22 *μ*m filter. Waters 2695-2996 was used to perform HPLC detection. The chromatographic column is Ultimate XB-C18, 4.6 × 250 mm, 5 *μ*m, FX-018; mobile phase was methanol: 0.1% phosphoric acid (13 : 87). The flow rate was 1.0 mL/min, and the column temperature was 35°C. The detection wavelength was 211 nm.

200 *μ*L of nude mouse blank plasma and each tissue homogenate was mixed with NCTD and was centrifuged at 10000 r/min for 5 min. 200 *μ*L of supernatant tissue homogenate was collected and was added with 3 mL methanol solution. After 10000 r/min centrifuge for 5 min, the supernatant was collected and was filtered with 0.22 *μ*m. Samples were detected by HPLC. The standard solution of plasma and each tissue was prepared according to the precision and standard curve gradient (0.004575 mg/mL, 0.022875 mg/mL, 0.045750 mg/mL, 0.09150 mg/mL, 0.137250 mg/mL). Each sample was injected 6 times in parallel [18].

### 2.7. Nude Mouse Tumorigenic Model

HepG2 was purchased from the National Experimental Cell Resource Sharing Platform and preserved in Kunming Medical University and Yunnan Key Laboratory of Natural Medicine Pharmacology. Twenty-eight 4-6-week-old male BALB/C nude mice were provided by the Experimental Animal Center of Kunming Medical University. After inoculation, 24 nude mice were randomly divided into the following 4 groups (6 mice in each group): tumor-bearing nude mice were injected with NCTD through tail vein, tumor-bearing nude mice were injected with NCTD-NLC through tail vein, tumor-bearing nude mice (gavage) NCTD, and tumor-bearing nude mice (gavage) NCTD-NLC. The remaining 4 were used as experimental death supplements. The hepatoma cells were subcutaneously inoculated in the right armpit of nude mice at 1 × 10^7^ cells per mouse. After tumor inoculation in nude mice, tumor growth was observed every other day for 3 weeks. When the tumor grows to about 0.5 cm^3^ in size, observe and measure the tumor with a vernier caliper every other day [19].

### 2.8. Statistical Analysis

In this study, GraphPad Prism 8.0 was used for data analysis, and *P* < 0.05 was considered statistically significant.

## 3. Results

### 3.1. NCTD-NLC Inhibited the Proliferation of Liver Cancer HepG2 Cells

**Figure 1:**
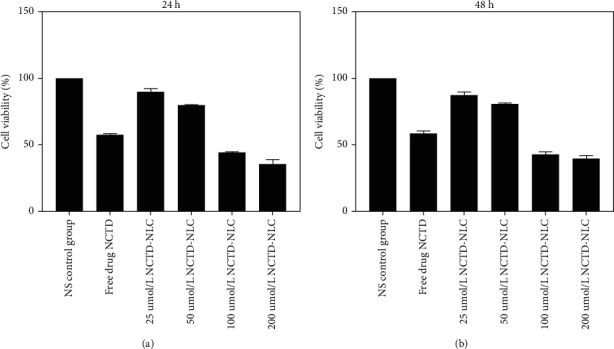
MTT detection of hepatoma cell proliferation after NCTD-NLC treatment. (a) 24 h after treatment in each group, MTT assay was conducted to estimate cell viability. (b) After 48 h treatment in each group, MTT assay was conducted to estimate cell viability.

For the purpose of clarifying the regulation of NCTD-NLC on the proliferation of liver cancer cells, MTT assay was performed to detect cell proliferation. Cells were divided to 6 groups: the NS control group, the free drug NCTD control group, 25 *μ*mol/L NCTD-NLC group, 50 *μ*mol/L NCTD-NLC group, 100 *μ*mol/L NCTD-NLC group and 200 *μ*mol/L NCTD-NLC group. The cell viability in each group was 100%, 57.59%, 90.09%, 79.77%, 44.22%, and 35.59%, respectively (Figure 1(a)). After 48 h of treatment, the cell viability in each group was 100%, 58.72%, 87.56%, 80.93%, 42.82%, and 39.44%, respectively (Figure 1(b)). It can be concluded that NCTD-NLC significantly inhibited cell viability and decreased viability of HepG2 cells. Cell viability was negatively correlated with NCTD-NLC concentration.

### 3.2. NCTD-NLC Promoted Apoptosis of HepG2 Cells

**Figure 2:**
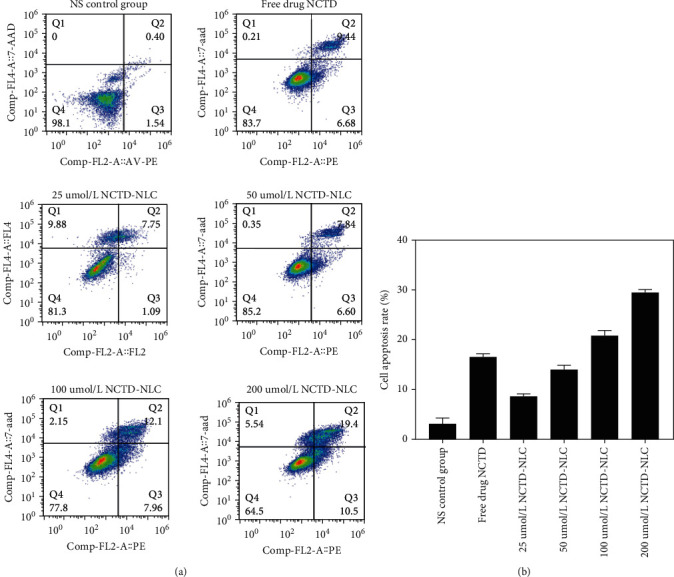
Flow cytometry assay to estimate cellular apoptosis: (a) flow cytometry results of apoptosis detection; (b) statistical results.

Next, flow cytometry was used to detect cell apoptosis. The results showed that the average apoptosis rates of those groups above were 3.12%, 15.56%, 8.66%, 4.05%, 20.82%, and 29.49%, respectively (Figure 2). After NCTD-NLC treatment, cell apoptosis was increased with the increase of concentration, and the viability was gradually decreased. The apoptosis rate was positively correlated with the concentration of NCTD-NLC.

### 3.3. After the SD Rats Were Administered, HPLC Was Used to Detect the Content of NCTD in the Heart, Liver, Spleen, Lung, Kidney, and Plasma of the Rats

**Figure 3:**
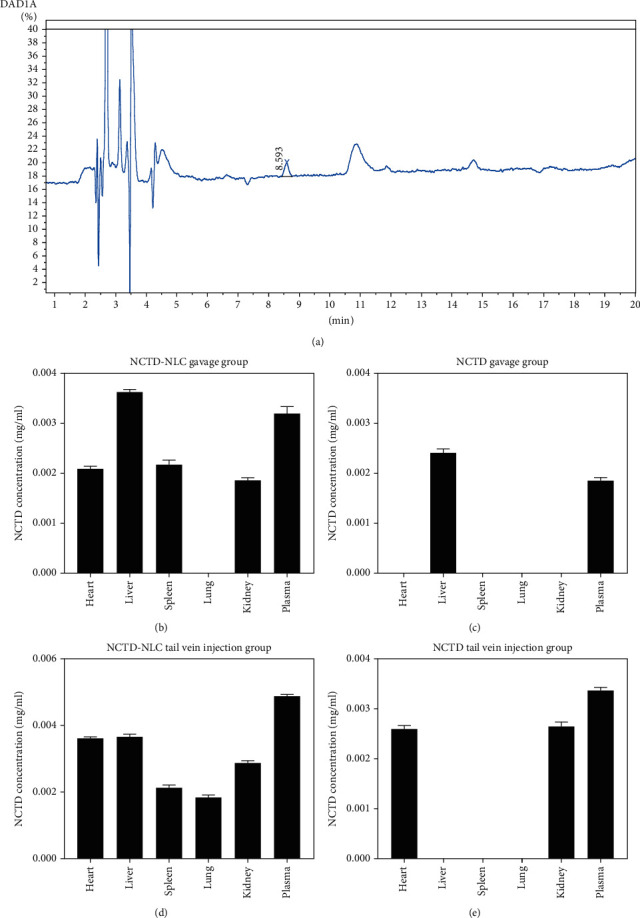
The HPLC detection onto the groups of NCTD-NLC gavage, NCTD gavage, NCTD-NLC tail vein injection, and NCTD tail vein injection to figure out the NCTD content in the heart, liver, spleen, lung, kidney, and plasma. (a) HPLC profiles. (b) The NCTD HPLC profiles of the heart, liver, spleen, lung, kidney, and plasma in the NCTD-NLC gavage group. (c) The NCTD HPLC profiles of the heart, liver, spleen, lung, kidney, and plasma in the NCTD gavage group. (d) The NCTD HPLC profiles of the heart, liver, spleen, lung, kidney, and plasma in the NCTD-NLC tail vein injection group. (e) The NCTD HPLC profiles of the heart, liver, spleen, lung, kidney, and plasma in the NCTD tail vein injection group.

The amount of NCTD in each organ was determined by HPLC (Figure 3(a)). The results showed that the NCTD was not detected in the heart tissue of NCTD gavage group; when compared to the other three groups, the NCTD-NLC tail vein injection group had significantly higher NCTD content (*P* < 0.001). No NCTD was detected in liver tissue in the NCTD tail vein group. The NCTD-NLC tail vein injection group had higher NCTD content than the NCTD-NLC gavage group, but the difference was not significant (*P* = 0.479). NCTD-NLC tail vein injection compared to the NCTD gavage group, the NCTD group had a higher content of NCTD, ending up with significant difference (*P* < 0.001). The NCTD was not detected in the spleen tissue of the NCTD gavage group and the NCTD tail vein group. The NCTD content of the NCTD-NLC tail vein injection group was lower than that of the NCTD-NLC gavage group but far from significantly different (*P* = 0.371). In the lung tissue, except for the NCTD-NLC tail vein injection group, there was no NCTD detected in other groups. Compared with the NCTD-NLC gavage group and the NCTD tail vein injection group, the content of NCTD in the NCTD-NLC tail vein injection group was increased, and the difference was significant (*P* < 0.001, *P* < 0.05). In the plasma samples, NCTD was detected in each group. When compared with the NCTD-NLC gavage group, NCTD gavage group, and NCTD tail vein injection group, the NCTD content of NCTD-NLC tail vein injection group increased, whose distinction was very significant (all *P* < 0.001) (Figure 3(b)).

### 3.4. Detecting the Inhibitory Effect of NCTD-NLC on HepG2 in the Nude Mouse Model

**Figure 4:**
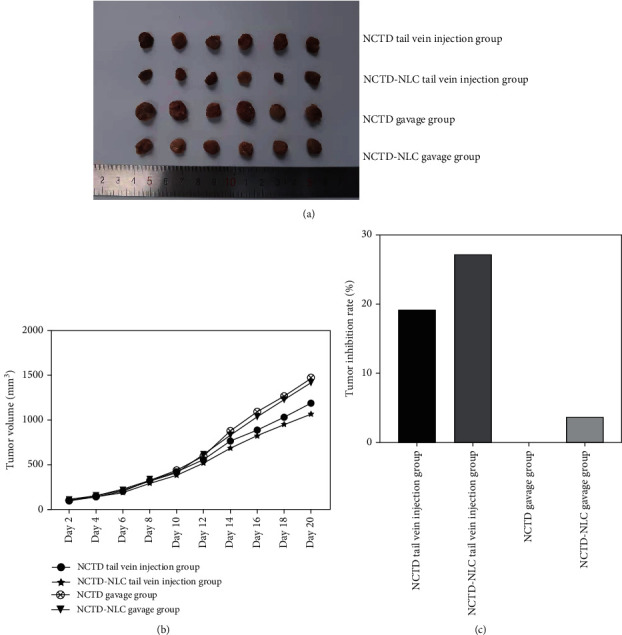
Results of tumor formation in nude mice after treatment in each group: (a) tumors isolated from each group of mice; (b) graph of tumor volume of nude mice in each group; (c) statistical results of tumor inhibition rate in each group.

The cultured hepatoma cells were inoculated under the armpit of nude mice. When our tumor-bearing model was successfully constructed and the tumor volume reached 0.5 cm^3^, the administration was started. On the 30th day of feeding, the nude mice of each group were sacrificed in groups, the tumor tissue was completely removed, and both the parameters of tumor mass and tumor volume were determined (Figures 4(a) and 4(b)). The results showed that compared with the NCTD gavage group, the tumor inhibition rate in the tail vein injection NCTD group was 19.15%, the tail vein injection NCTD-NLC group was 27.48%, and the NCTD-NLC gavage group was 3.77% (Figure 4(e)). The above results indicated that the tail vein injection of NCTD-NLC had the best inhibitory effect on tumors.

### 3.5. Detection of NCTD Content in Tumor by HPLC

**Figure 5:**
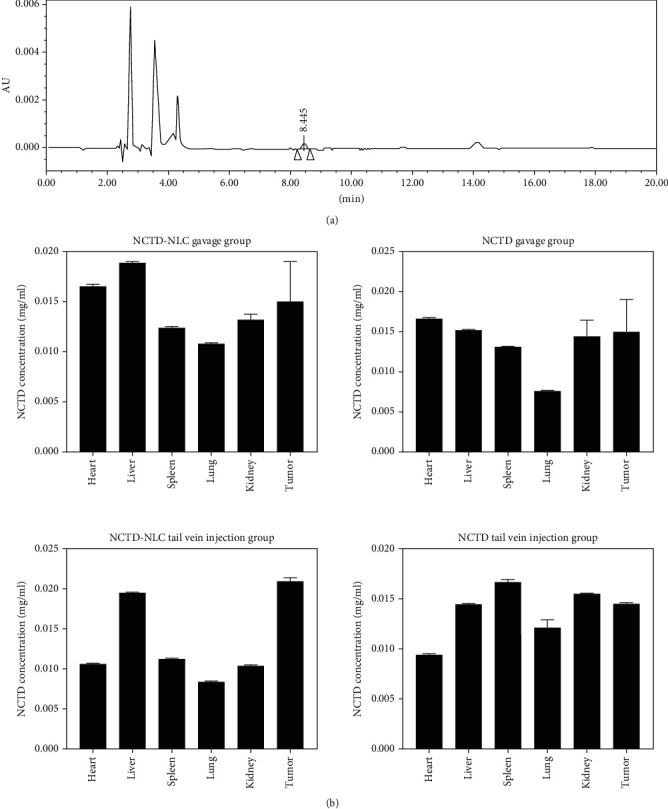
Detection of NCTD content in tumors by HPLC: (a) HPLC profiles of NCTD; (b) statistical results of NCTD content.

Next, we further used HPLC to detect the content of NCTD in tumor tissues of each group. The results showed that NCTD was found in the tumor tissues of each group. The NCTD content in the NCTD-NLC tail vein injection group was significantly higher than that in both NCTD gavage group and NCTD tail vein injection group (all *P* < 0.001). In the comparison of the NCTD-NLC gavage group, the content of NCTD in the NCTD-NLC tail vein injection group increased, but the difference was not significant (*P* = 0.740) (Figure 5).

## 4. Discussion

Chemotherapy mainly relies on drug toxicity to slow down the malignant proliferation of tumor cells, but it also suppresses the proliferation of hair follicles and bone marrow, resulting in hair loss, bone marrow suppression, nausea and vomiting, and other side effects [20]. Although chemotherapy drugs and chemotherapy methods have been greatly changed in the past few decades, the above toxic and side effects are still unavoidable. Therefore, traditional Chinese medicine ingredients were selected as model drugs.

In recent years, increasing evidence of studies has shown that cantharidin has anticancer properties in various cancers, comparing lung, gastric, colorectal, pancreatic, and bladder cancers. Studies have shown that NCTD has an effect towards non-small-cell lung cancer. NCTD can inhibit signal transduction in lung cancer cells to hinder both cellular growth and migration and can also promote cell autophagy and apoptosis, showing anticancer activity against lung cancer [21]. NCTD is an ideal candidate to non-small-cell lung cancer treatment. Some studies have used cantharidin pegylated liposomes to explore its therapeutic effect on liver cancer in vitro and in vivo and evaluated its anticancer efficiency in vivo in nude mice [22]. The outcomes demonstrated that the new formulation significantly enhanced the tumor suppressive effect of NCTD. The results of this study showed that cell viability of HepG2 cells was significantly inhibited by NCTD-NLC. Cell viability was negatively correlated with NCTD-NLC concentration. The results of this study are consistent with previous results, indicating that NCTD can inhibit tumor cell proliferation and viability.

In addition to the described anticancer effects, cantharidin also exhibits the advantage of lower drug resistance, which is especially important when multidrug resistance is a common problem in anticancer therapy [23, 24]. In order to improve the bioavailability of oral NCTD, it was prepared as a solid lipid carrier, and the free drug and nanoparticles have been assessed in vitro and in vivo, and the pharmacokinetics were evaluated. NCTD, which is effective against colorectal cancer cells, is encapsulated in nanoparticles of a novel drug delivery system to reduce its side effects and enhance its efficacy [25, 26]. Cantharidin has attracted much attention as an effective anticancer ingredient, but studies have shown that cantharidin and its analogs such as NCTD have been shown to be effective in those nonsolid tumors like chronic myeloid leukemia and acute myeloid leukemia. In this study, the flow cytometry results also indicated that the cellular apoptosis of HepG2 treated with NCTD-NLC was significantly advanced with the increase of the concentration. The apoptosis rate was positively correlated with the concentration of NCTD-NLC. The modified NCTD treated liver cancer cells with a higher apoptosis rate, indicating that the modified NCTD has better tumor suppressor effect.

The tumor “EPR effect” may be an effective method for nanostructured lipid carriers to achieve NCTD targeting. Tumor tissue has abnormal vasculature (excessive vascularization) with large gaps in poorly aligned and defective endothelial cells, so that nanodrug delivery systems can passively aggregate tumor tissue, which is referred to as the tumor “EPR effect” (enhanced penetration and retention effect) [27]. The tumor “EPR effect” of nanodrug delivery systems is closely related to the composition, size, surface charge, and administration route of nanodrug delivery systems [28]. It was found that due to the rapid growth of tumors, discontinuous capillaries, and small interstitial windows, nanoparticles can easily overflow from blood vessels and reach the tumor stroma, so that they can accumulate in the tumor site and can also be carried out in the extracellular space. 60-200 nm nanodrug delivery systems are able to infiltrate tumor blood vessels and stagnate in tumor tissues instead of in normal tissues [29]. This NCTD-NLC prepared with the particle size of less than 200 nm in this study has a neutral charge on the surface, relying on the tumor “EPR effect” to passively aggregate to tumor tissue. The strategy of passive targeting is endowed with the merits of less toxicity and few side effects to a certain extent, improving the specific therapeutic effect on tumors. In the comparison of small-molecule chemical medicines, nanostructured lipid carriers are featured with unique tumorous aggregation because of their preferential tumor-site distribution. It can also help to kill cancer cells entering the blood by maintaining and prolonging the plasma drug concentration, preventing the metastasis of cancer cells, and indirectly increasing the aggregation of particulate drugs in target tissues.

Herein, we demonstrated that NCTD-NLC functioned in a superior tumor suppressor manner in both in vitro and in vivo experiments. Meanwhile, we measured the NCTD content in each tissue of tumor-bearing mice, whose results unveiled that the content in tumors were significantly increased, verifying that NCTD-NLC may depend the tumor “EPR effect” exerted passive aggregation, and the higher drug delivery effect of NCTD-NLC was also compared by HPLC. The results of this study provide new ideas and research foundations for more effective utilization of NCTD in the future. The limitation of this study is that it failed to deeply explore the target of NCTD and failed to use molecular docking or coprecipitation studies to clarify the molecular regulation mechanism of NCTD. Most of the content was the detection of phenotypes. Therefore, in future research, in order to further promote the clinical application of NCTD, we will further explore the molecular regulation mechanism.

## 5. Conclusions

In conclusion, the NCTD-NLC prepared in this study is capable of significantly alleviating the viability of HepG2 cells and promoting their apoptosis. Tail vein injection of NCTD-NLC has the best drug delivery effect.

## Data Availability

The data used to support the findings of this study are included within the article.
